# Bias and Loss to Follow‐Up in Cardiovascular Randomized Trials: A Systematic Review

**DOI:** 10.1161/JAHA.119.015361

**Published:** 2020-07-09

**Authors:** Lucas Chun Wah Fong, Thomas J. Ford, Bruno R. da Costa, Peter Jüni, Colin Berry

**Affiliations:** ^1^ West of Scotland Heart and Lung Centre Golden Jubilee National Hospital Glasgow Scotland; ^2^ British Heart Foundation Glasgow Cardiovascular Research Centre Institute of Cardiovascular and Medical Sciences University of Glasgow United Kingdom; ^3^ Department of Cardiology Gosford Hospital Gosford NSW Australia; ^4^ Faculty of Medicine University of Newcastle Callaghan NSW Australia; ^5^ Department of Medicine University of Toronto Canada; ^6^ Institute of Health Policy, Management, and Evaluation Dalla Lana School of Public Health University of Toronto; ^7^ Applied Health Research Center (AHRC) Li Ka Shing Knowledge Institute, St Michael's Hospital Toronto Ontario Canada; ^8^ Institute of Primary Health Care (BIHAM) University of Bern Bern Switzerland

**Keywords:** bias, loss to follow‐up, outcome, outcome and process assessment, patient dropout, randomized controlled trials, relative risk, Mortality/Survival, Statements and Guidelines, Meta Analysis

## Abstract

**Background:**

Loss to follow‐up (LTFU) is common in randomized controlled trials. However, its potential impact on primary outcomes from cardiovascular randomized controlled trials is not known.

**Methods and Results:**

We conducted a prospective systematic review (PROSPERO: CRD42019121959) for randomized controlled trials published in 8 leading journals over 5 years from January 2014 to December 2018. Extent, reporting, and handling of LTFU data were recorded, and the proportion of a trial's primary outcome results that lose statistical significance was calculated after making plausible assumptions for the intervention and control arms. These assumptions could drive differential treatment effects between the groups considering relative event incidence between LTFU participants and those included in the primary outcome. We identified 117 randomized controlled trials of which 91 (78%) trials reported LTFU, 23 (20%) reported no LTFU, and 3 (3%) trials did not report on whether LTFU occurred. The median percentage of study participants lost to follow‐up was 2% (interquartile range, 0.33%–5.3%). Only 10 trials (9%) had a low cluster of risk factors for impairment in trial quality. The percentage of trials losing statistical significance varied from 2% when the relative event incidence for LTFU between the randomized groups was 1 for the intervention arm and 1.5 for the control arm to 16% when the relative event incidence was 3 for the intervention arm and 1 for the control arm.

**Conclusions:**

Almost 1 in 6 (16%) cardiovascular randomized trials published in leading journals may have a change in the primary outcome if plausible assumptions are made about differential event rates of participants lost to follow up. There is scope for improvement arising from LTFU in randomized trials in cardiovascular medicine.

**Registration:**

URL: https://www.crd.york.ac.uk/prospero; Unique identifier: CRD42019121959.

**Nonstandard Abbreviations and Acronyms**
LTFU: loss to follow‐up
RCT: randomized controlled trial
RI: relative event incidence among those lost to follow‐up to the event incidence among those followed up

**Clinical Perspective**

**What Is New?**

- More than three quarters of cardiovascular randomized controlled trials have participants who are lost to follow‐up. Statistical handling of these data vary widely.
- Up to 1 in 6 trials may have a change in the primary outcome if plausible assumptions are made about differential event rates of participants lost to follow up.

**What Are the Clinical Implications?**

- In dealing with loss to follow‐up (LTFU), prevention should be prioritized; otherwise, estimation can be made by using the worst assumption.
- When reporting LTFU, authors should provide baseline characteristics of LTFU participants, extent of follow‐up before exclusion, and time of dropout and should address implication of LTFU when interpreting results.
- Inadequate allocation concealment is an independent factor associated with LTFU and may drive differential treatment effects.

The gold standard assessment of a medical intervention involves assessment in a randomized controlled trial (RCT).^1^, ^2^ Randomization balances the distribution of any known or unknown potential confounding factors between treatment arms.^2^ This mitigates the possibility of selection bias, especially if the participants’ group allocations are concealed.^3^ Blinding of patients, therapists, and outcome assessors is an additional useful tool to prevent bias.^4^, ^5^ Open‐label clinical trials are often unavoidable if blinding of patients and therapists is not possible.^6^ Clinical guidelines may be influenced by biased clinical evidence leading to undesirable impacts on patients, healthcare providers, and funders.

Up to 80% of contemporary clinical RCTs fail to achieve complete follow‐up.^7^, ^8^, ^9^ This important factor may affect the integrity of study conclusions. If participants are lost and the characteristics of such participants associate with clinical events, then bias can arise. This is particularly relevant in open‐label studies in which assessors know the group allocations of the participants. Loss to follow‐up (LTFU) in this scenario could favor the intervention arm and neutralize the benefit of randomization.^10^ It is plausible that attrition bias associated with LTFU drives either overestimation or underestimation of treatment effects.^11^, ^12^

Classification of LTFU and recommendations for dealing with LTFU have been made.^13^ Crucially, however, the contemporary prevalence and effects of LTFU within cardiovascular trials is not known. This prospective systematic review and meta‐analysis was designed to analyze the prevalence and potential impact of LTFU in cardiovascular RCTs. The primary aim was to assess the proportion of trials in which the primary efficacy end point would change if plausible assumptions were made about participants who were unaccounted for in the original analysis. In addition, we assessed estimates of treatment effect according to the extent, reporting, and handling of LTFU and trial characteristics associated with LTFU.

## Methods

### Eligibility

All supporting data are available within the article and its online supplementary files. Ethics approval was not required. We predefined reports as being eligible for inclusion in this analysis if an RCT in cardiovascular disease was described and published in one of the 5 leading general medical journals and 3 cardiology journals with the highest impact factors (*Annals of Internal Medicine, BMJ, JAMA, Lancet, New England Journal of Medicine, Circulation, European Heart Journal*, and *Journal of the American College of Cardiology*). A 5‐year publication period was set from 2014 to 2018. An additional inclusion criterion was if a patient‐important binary primary outcome was statistically significant at a 2‐sided α of 0.05. The rationale behind focusing on statistically significant trials in major journals only is that the results of these trials are most likely to influence clinical guidelines. Therefore, a change in significance of a risk ratio due to bias might affect patient care to an important extent. Cluster trials, crossover trials, N‐of‐1 trials, and trials reported in research letters were excluded. Equivalence and noninferiority studies were excluded unless the authors prespecified testing for superiority. Reports describing secondary analyses of randomized trials were excluded.

A patient‐important outcome was defined as an outcome that would be undesirable for a patient to experience and the patient would thus try to prevent it by undergoing an effective treatment. Mortality and morbidity are examples of outcomes that were included. Surrogate outcomes were considered as nonpatient important (Data S1). The protocol was registered on PROSPERO (CRD42019121959).

### Literature Search

Reports of RCTs were identified from Medline and Embase using OVID (Data S2). The search was restricted to clinical RCTs in cardiovascular disease published in the selected journals between 2014 and 2018. Trials were considered statistically significant if the 2‐sided 95% CI of an estimate of the relative risk did not include 1.0 or if the 2‐sided *P* value for superiority was <0.05 when no CI was reported. A calibration exercise was performed before the search. One reviewer identified and reviewed the potentially eligible reports based on an agreed screening form (Data S3). The list of included and excluded reports was provided to the 2‐person reviewer team after screening. Disagreements were resolved by consensus, with the assistance of a third reviewer as required.

### Data Collection

Data were extracted based on an agreed data extraction form (Data S4). The primary outcome selected for the review was the one specified within the report. If the report specified both significant primary efficacy and safety outcomes, the primary efficacy outcome was selected. If multiple primary outcomes were specified, the statistically significant outcome in the highest category on the outcome hierarchy was selected (Data S1). If both intention‐to‐treat and per‐protocol analyses were reported, we considered the statistical significance of the former; if both unadjusted and adjusted analyses were reported, the statistical significance of the former was considered. Data on study background, general characteristics, methodological quality,^14^ the extent of LTFU, its reporting, and its handling in the analysis related to the primary outcome were extracted. Patients were considered as LTFU if they were mistakenly randomized with inappropriate postrandomization exclusion; did not receive the intervention or adhere to treatment, with inappropriate postrandomization exclusion; withdrew consent; crossed over to another arm but were not included in the analysis; or lost contact.^15^ Trials were categorized by subspecialty focus: electrophysiology, heart failure, interventional cardiology, cardiac surgery, general cardiology, and cardiovascular imaging.

### Statistical Analysis

The analysis is explained in more detail in the online supplement (Data S5). Methodological and reporting quality of the included trials was assessed, as suggested by Bikdeli et al^14^ and the Cochrane risk‐of‐bias assessment tool.^16^ The extent of LTFU was calculated as the percentage of LTFU in each trial from each arm (intervention and control). The ratio of LTFU rate to primary event rate was also reported. A univariable random‐effects metaregression analysis was conducted using the log odds of participants lost to follow‐up as the dependent variable and general trial characteristics and methodological characteristics as independent variables.

The potential impact of LTFU on the primary outcome analysis was evaluated by making assumptions about the outcomes in LTFU participants (Data S6). An estimation algorithm proposed by Akl et al^17^ was adopted with relative incidence of outcomes in LTFU patients compared with patients who were followed‐up (RI_LTFU/FU_), ranging from 1 to 3.^16^ In addition, the following common assumptions were used for calculations: none of the participants lost to follow‐up had the event; all participants lost to follow‐up had the event; none of those lost to follow‐up in the treatment group had the event, and all those lost to follow‐up in the control group did (best case scenario); all participants lost to follow‐up in the treatment group had the event and none of those in the control group did (worst‐case scenario).

For each trial, 2×2 tables were constructed for the collected data for the calculation of risk ratios associated with each assumption. The percentage of trials with their primary outcome becoming nonsignificant was calculated based on the assumptions and definition of statistical significance reported above. Trials with no LTFU were excluded in the primary analysis but included in a sensitivity analysis. An additional prespecified sensitivity analysis stratified by type of intervention was conducted. Paired differences in proportions between interventional cardiology trials and those of other cardiology subspecialties were also assessed based on different assumptions.

## Results

**Figure 1:**
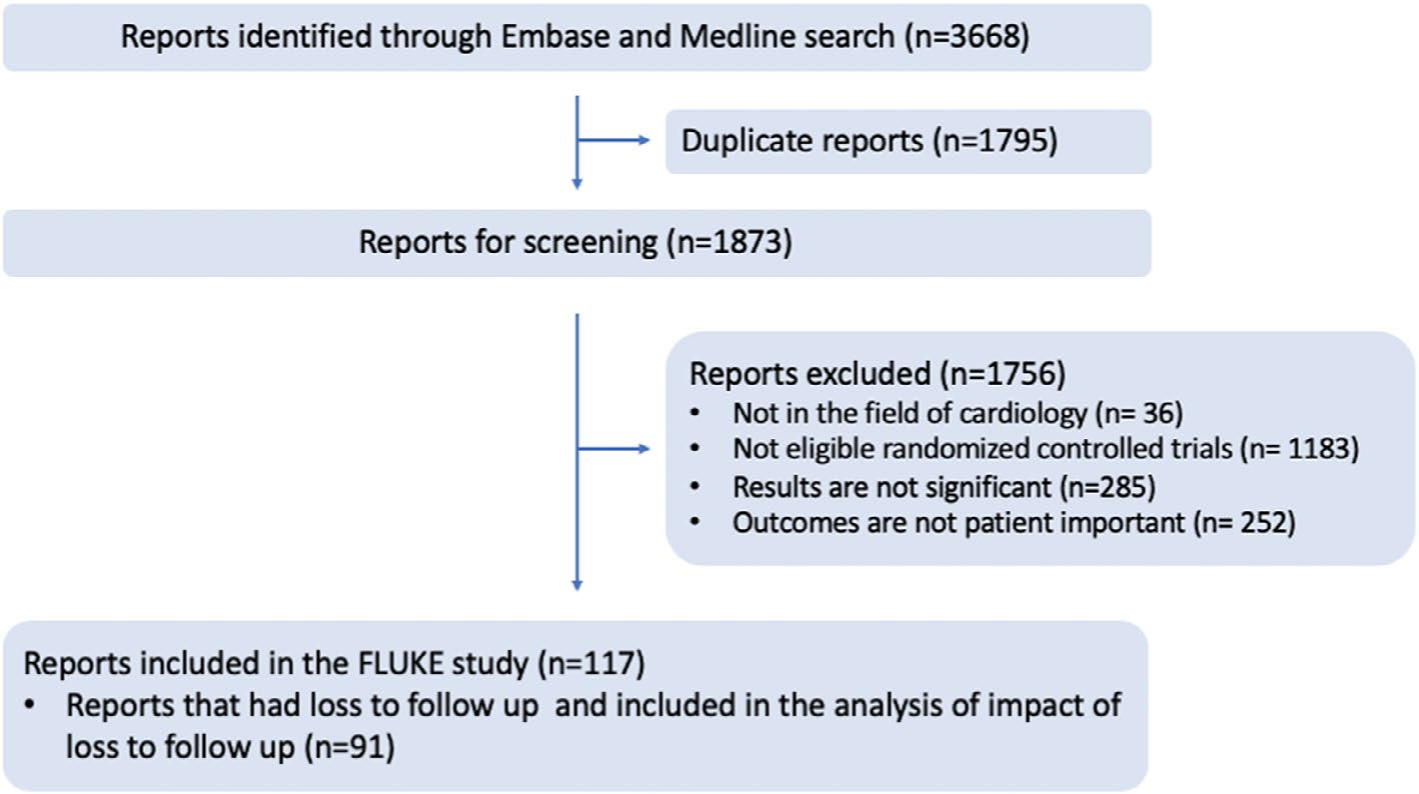
Search and screening approach. Flow of trial reports identified and screened in this analysis is shown. The search recovered 3668 reports; 1873 reports were screened after removing duplicates; 117 reports were included after screening, and reasons for exclusion are stated in text. FLUKE indicates Follow Up Loss Effect Upon Skewing Evidence.

After excluding duplicates and screening for eligibility, 117 studies were included from a total of 3668 from the initial search (Figure 1). The list of the 117 studies included in this analysis is provided in Table S1.^18^, ^19^, ^20^, ^21^, ^22^, ^23^, ^24^, ^25^, ^26^, ^27^, ^28^, ^29^, ^30^, ^31^, ^32^, ^33^, ^34^, ^35^, ^36^, ^37^, ^38^, ^39^, ^40^, ^41^, ^42^, ^43^, ^44^, ^45^, ^46^, ^47^, ^48^, ^49^, ^50^, ^51^, ^52^, ^53^, ^54^, ^55^, ^56^, ^57^, ^58^, ^59^, ^60^, ^61^, ^62^, ^63^, ^64^, ^65^, ^66^, ^67^, ^68^, ^69^, ^70^, ^71^, ^72^, ^73^, ^74^, ^75^, ^76^, ^77^, ^78^, ^79^, ^80^, ^81^, ^82^, ^83^, ^84^, ^85^, ^86^, ^87^, ^88^, ^89^, ^90^, ^91^, ^92^, ^93^, ^94^, ^95^, ^96^, ^97^, ^98^, ^99^, ^100^, ^101^, ^102^, ^103^, ^104^, ^105^, ^106^, ^107^, ^108^, ^109^, ^110^, ^111^, ^112^, ^113^, ^114^, ^115^, ^116^, ^117^, ^118^, ^119^, ^120^, ^121^, ^122^, ^123^, ^124^, ^125^, ^126^, ^127^, ^128^, ^129^, ^130^, ^131^, ^132^, ^133^, ^134^ The mean age of 407 229 study participants was 64.2 years (30% female). The trial subspecialties were electrophysiology (19%) heart failure (3%), interventional cardiology (28%), cardiac surgery (3%), general cardiology (44%), and cardiovascular imaging (3%). Baseline study characteristics of the included trials are reported in Table 1.

**Table 1:** General Characteristics of 117 Included Trials in the Study (n=117)

	No. (%)
Extent of loss to follow‐up (overall)	
<1%	34 (38)
1% to ≤2.5%	19 (21)
2.5 to ≤5%	14 (15)
5% to ≤7.5%	9 (10)
7.5% to ≤10%	3 (3)
>10%	12 (13)
Cardiology subspecialty	
Electrophysiology	22 (19)
Heart failure	3 (3)
Interventional cardiology	33 (28)
Open heart surgery	4 (3)
General cardiology^a^	51 (44)
Cardiovascular imaging	4 (3)
Control	
Standard care	18 (15)
Placebo	31 (27)
Pharmacological	28 (24)
Surgical/interventional	36 (31)
Other	4 (3)
Funding	
Private for profit	58 (50)
Private not for profit	21 (18)
Governmental	24 (20)
Not reported	13 (11)
Not funded	1 (1)
Reporting of methods to deal with LTFU	
Reported in methods	100 (86)
Reported in results	1 (1)
No	16 (14)
Among the trials that LTFU occurred (n=91)^b^	
Separately reported in 2 arms	70 (77)
Compared the LTFU group baseline characteristics with not LTFU	0 (0)
Implication of LTFU discussed	6 (7)
Analytical method to handle LTFU	
No LTFU occurred	26 (22)
Complete case analysis^c^	10 (8)
Worst‐case scenario	2 (2)
Multiple imputation	2 (2)
Inverse probability weighting	0 (0)
Censored at time of LTFU in time‐to‐event analysis	75 (64)
Assumption that none of the LTFU participants have event	2 (2)
CONSORT diagram	
Without the diagram	32 (27)

CONSORT indicates Consolidated Standards of Reporting Trials; LTFU, loss to follow‐up.

a General cardiology trials in this review referred to pharmacological trials and lifestyle‐changing trials.

b Number shown refers to trials that did the following.

c Complete case analysis is defined as an analysis that only include patients with complete outcome data. LTFU patients are excluded from the whole analysis.

### Assessment of the Methodological Quality of the Trials

The analytical methods that were used for handling LTFU in the primary analysis of the included trials are presented in Table 1. The most commonly used method was censoring at time of LTFU in a time‐to‐event analysis (N=75; 64%). Two trials (2%) assumed that no LTFU participants experienced events, whereas 10 (8%) used complete case analysis and 2 (2%) used a worst‐case scenario in which only the control arm had events. Two trials (2%) used multiple imputation, whereas none reported using inverse probability weighting.

Regarding the reporting of LTFU, 85 (73%) used a Consolidated Standards of Reporting Trials (CONSORT) diagram. Seventy (77%) trials reported that LTFU occurred in the intervention and control arms separately. However, none of the trials compared baseline characteristics of LTFU participants with followed‐up participants. The implications of LTFU are discussed in 6 trials (7%).

**Figure 2:**
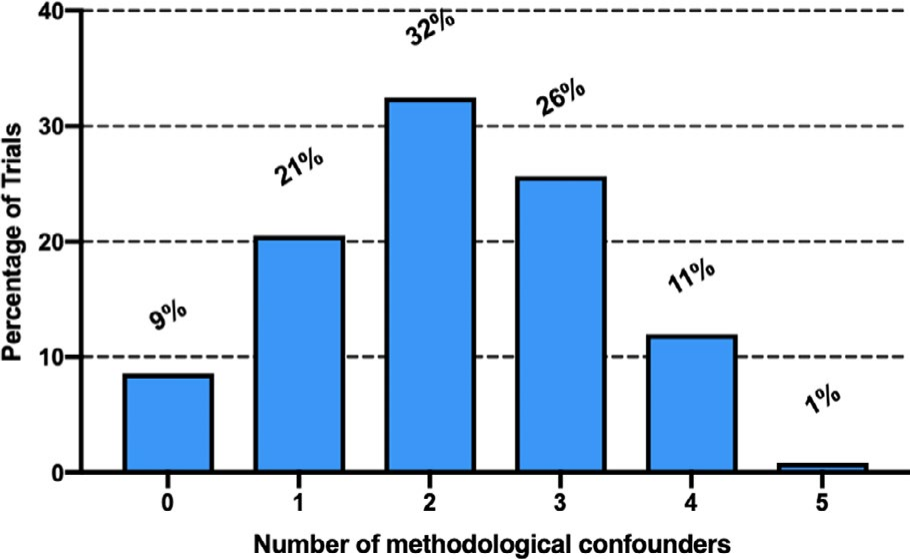
Distribution of trials according to methodological and reporting quality assessments that might impair the outcomes of the trial. Distribution of trials according to the number of methodological and reporting quality characteristics (methodological confounders) they possess after the assessment: 9% of the trials had none of the methodological confounders (n=10), 21% of the trials possessed 1 methodological confounder (n=24), 32% of the trials possessed 2 major methodological confounders (n=38), and 26% of the trials possessed 3 major methodological confounders (n=30). In addition, 12% of the trials had >3 methodological confounders. This list of methodological confounders analyzed included the following: (1) inadequate allocation sequence concealment, (2) no blinding of patients, (3) early stop of trial, (4) not using intention‐to‐treat analysis, (5) absence of protocol, and (6) no explicit statement about status of loss to follow‐up.

Table 2 and Figure 2 demonstrated the number of trials meeting the characteristics (methodological confounders) for impairment in the quality of trial design. Allocation concealment was adequate in 54 trials (46%). Patients were blinded adequately in 41 trials (35%). In 9 trials (8%), enrollment was discontinued prematurely. Twenty‐nine trials used an intention‐to‐treat analysis (25%). Thirty‐one trials (26%) provided a protocol. Forty‐three trials (36%) did not state the status of LTFU explicitly in the report. Only 10 trials (9%) were free from any methodological confounders that might impair the methodological quality.

**Table 2:** Methodological and Reporting Quality Assessment of the Included Trials

Factors	Trials at Risk of Bias (n=117), No. (%)
Inadequate allocation sequence concealment^a^	63 (54)
No blinding of patients^b^	76 (65)
Early stop	9 (8)
Not using intention‐to‐treat analysis^c^	29 (25)
Absence of protocol^d^	31 (26)
Without explicit statement about status of LTFU	43 (36)

LTFU indicates loss to follow‐up.

a Allocation concealment defined as to the person enrolling participants does not know in advance which treatment the next person will get which usually involves the use of computer algorithms. It seeks to prevent selection bias by protecting the assignment sequence until allocation, and can always be successfully implemented.^136^ It is considered to be adequate according to the definition reported by Jüni et al.^3^

b Blinding defined as to the withholding information about the assigned interventions from people involved in the trial who may potentially be influenced by this knowledge; blinding is performed to prevent performance and ascertainment bias by protecting the sequence after allocation and cannot always be implemented.^136^, ^137^ It is considered to be adequate only if clearly indicated.

c Intention to treat analysis defined as an analysis that included all randomized participants in the analysis who are all retained in the group to which they were allocated.^3^, ^136^

d Consider as absence if the protocol is not published before or is included as appendix beside the main report.

Random‐effects metaregression analysis (Table 3) suggested that inadequate or unclear concealment of allocation was associated with an increase in odds of LTFU (odds ratio, 2.37 [95% CI, 1.42–3.97]; *P*=0.001). Increasing sample size (*P*=0.039) and duration of follow‐up (*P*=0.002) also increased the odds of LTFU. Finally, the odds of LTFU was decreased in nonsurgical or noninterventional trials (odds ratio, 0.50 [95% CI, 0.30–0.85]; *P*=0.01).

**Table 3:** Regression Analysis Exploring the Association Between the Percentage of LTFU Participants and General and Methodological Trial Characteristics

Trial Characteristic	No. of Trials (n=117)	No. of Patients	Odds Ratio (95% CI)	*P* for Interaction
Number of centers				0.098
1	17	57 048	1.00 (Reference)	
2–10	26	19 680	1.43 (0.56–3.65)	
11–50	34	60 940	2.24 (0.94–5.37)	
>50	40	259 600	1.99 (0.86–4.57)	
Sample size				0.039
≤500	43	10 971	1.00 (Reference)	
>500–1000	25	17 722	0.97 (0.45–2.05)	
>1000–5000	26	53 077	0.74 (0.36–1.51)	
>5000	23	315 498	0.50 (0.24–1.02)	
Concealment of allocation				0.001
Yes	54	153 572	1.00 (Reference)	
No	63	243 696	2.37 (1.42–3.97)	
Blinding of patients				0.15
Yes	41	288 784	1.00 (Reference)	
No	76	108 484	1.49 (0.86–2.59)	
Intention to treat				0.89
Yes	88	347 413	1.00 (Reference)	
No	29	49 855	1.04 (0.57–1.90)	
Length of follow‐up, mo				0.002
≤6	27	30 234	1.00 (Reference)	
>6 to 12	35	50 278	1.89 (0.87–4.10)	
>12 to 24	26	67 459	2.53 (1.12–5.72)	
>24	29	249 297	3.42 (1.57–7.42)	
Trial stopped early				0.75
Yes	9	66 577	1.00 (Reference)	
No	108	330 691	1.16 (0.46–2.90)	
Surgery or interventional treatment				0.010
Yes	52	51 574	1.00 (Reference)	
No	65	345 694	0.50 (0.30–0.85)	
General cardiology				0.84
Yes	51	335 302	1.00 (Reference)	
No	66	61 966	1.06 (0.63–1.77)	
Commercial funding				0.78
Yes	58	299 427	1.00 (Reference)	
No	59	97 841	1.08 (0.63–1.83)	

LTFU indicates loss to follow‐up.

### Extent of Loss to Follow‐Up

**Figure 3:**
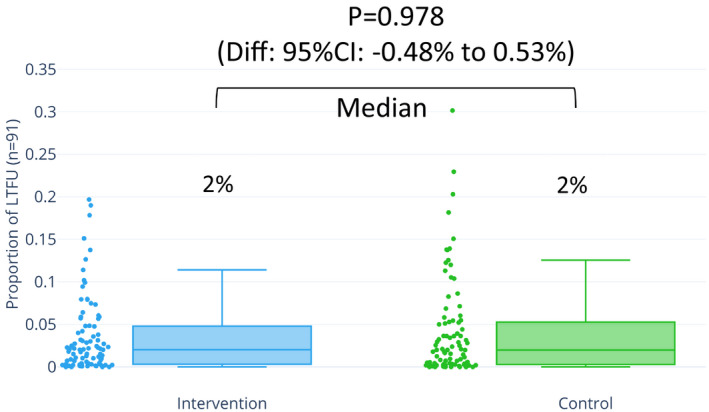
Distribution and difference in proportion of LTFU between the intervention and control arms among 91 trials with LTFU. Distribution of LTFU proportions among 91 trials that reported LTFU stratified by intervention and control. A median of 2% LTFU occurred in both the intervention and control arms. The difference is not significant (95% CI, −0.48% to 0.53%; *P*=0.978). Diff indicates difference; LTFU, loss to follow‐up.

Among the 117 included trials, 91 (78%) reported LTFU. Twenty‐three trials reported no LTFU (20%), and 3 trials did not report whether there was LTFU (3%). Of the trials with LTFU, the median percentage of LTFU was 2% (interquartile range [IQR], 0.3%–4.8%) in the intervention arm, 1.99% (IQR, 0.3%–5.4%) from the control arm, and 1.96% (IQR, 0.33%–5.3%) overall. The median difference between the intervention and the control groups was not significant (*P*=0.978; Figure 3).

**Figure 4:**
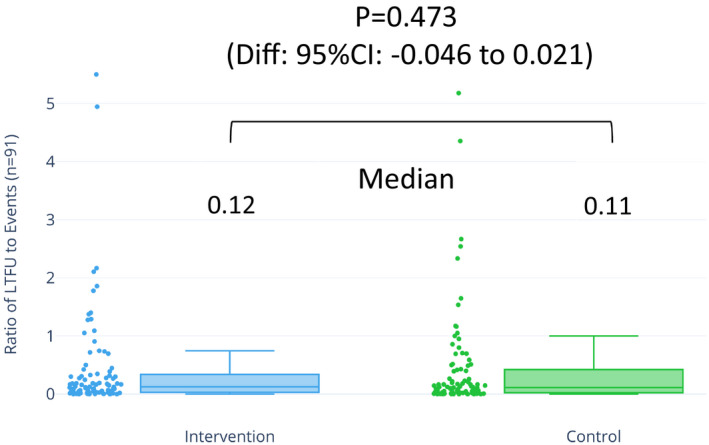
Distribution and difference in ratio of LTFU to events between the intervention and control arms among 91 trials with LTFU. Distribution of ratios across 91 trials with LTFU stratified by intervention and control. Medians of 0.12 from intervention arms and 0.11 from control arms indicate that ≈1 person was lost when 10 experienced events in both intervention and control arms. This shows the relativeness of proportions in between LTFU and events. The difference in ratio was not significant (95% CI, −0.046 to 0.021; *P*=0.473). Diff indicates difference; LTFU, loss to follow‐up.

The medians for the ratios of LTFU to events were 0.12 (IQR, 0.03–0.33) in the intervention arm, 0.11 (IQR, 0.02–0.42) in the control arm, and 0.11 (IQR, 0.03–0.41) overall. A value of 0.12 means that ≈1 participant is LTFU when every 10 participants experience the primary outcome. However, the difference between the ratio of the intervention and the control groups was not significant (*P*=0.473; Figure 4).

### Potential Impact of LTFU

#### Percentage of Trials Losing Significance

For the 4 common assumptions in which all 91 trials were included, the percentages of trials that lost significance were 4% (no participants lost to follow‐up had the event), 11% (all participants lost to follow‐up had the event), 3% (best‐case scenario), and 33% (worst‐case scenario).

**Figure 5:**
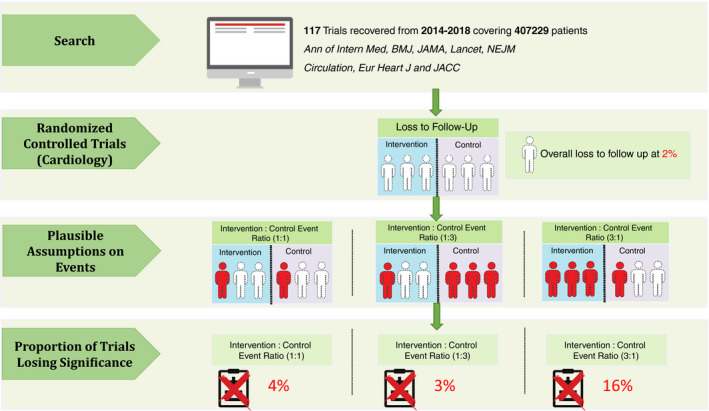
Bias and loss to follow‐up in randomized controlled trials in cardiovascular medicine. Assumptions being made toward the outcome of LTFU in each trial from the search and the subsequent calculation made. In total, 117 trials from 8 journals covering 407 229 patients from 2014 to 2018 were recovered. Assume participants were randomized to intervention and control, respectively; 3 had events from each arm and 3 dropouts from each arm. From the figure, dotted transparent figures denote LTFU participants, whereas red dotted figures denote LTFU participants being assumed with event. The plausible assumptions being made toward the LTFU was based on relative event incidence and a formula detailed in Data S6. The number of events were assumed based on the reported formula with incidence ranging from 1 to 3. Calculations of how many trials’ relative risks lost significance after making the assumptions were run subsequently. Ann of Intern Med, *Annals of Internal Medicine*; Eur Heart J, *European Heart Journal*; JACC,* Journal of the American College of Cardiology*; LTFU, loss to follow‐up; NEJM,* New England Journal of Medicine*.

**Figure 6:**
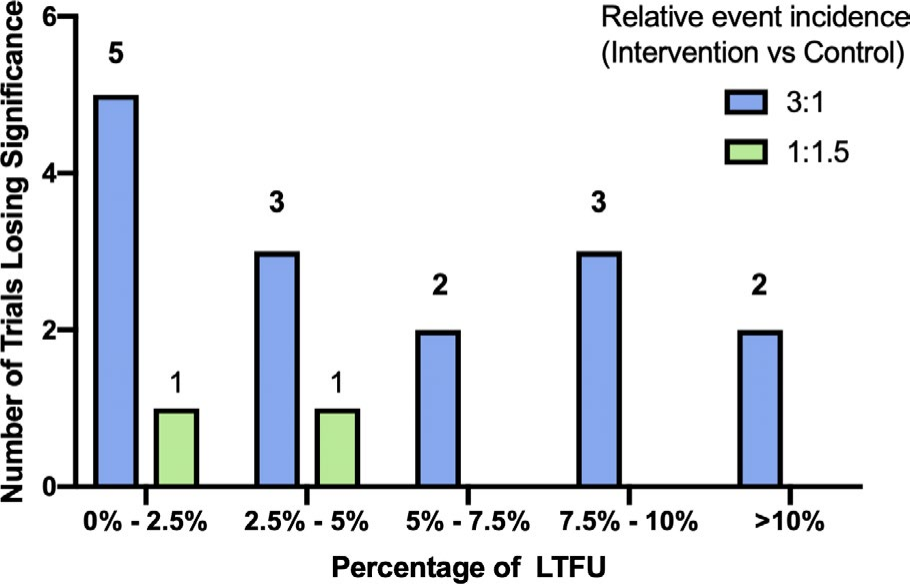
Distribution of trials by LTFU proportion under the best and worst plausible assumptions made by using the relative event incidence for the control and intervention arms. Distribution of trials losing statistical significance stratified by the percentage of LTFU of the individual trial under the best and worst assumptions made by the more plausible relative event incidence method. An inverse‐proportion relationship is shown in the graph, where there is higher number of trials losing significance in trials with lower proportions of LTFU. LTFU indicates loss to follow‐up.

Considering the relative event incidence analysis method, Table 4 shows the percentage of eligible trials that lost significance across a range of assumptions for the event incidence among intervention and control arms (Figure 5). The percentage varied from 2% to 16%. Figure 6 shows an inverse‐proportion relationship of the trials losing significance with the percentage of LTFU under the best and worst assumptions made by the relative event incidence analysis method.

**Table 4:** Percentage of 91 Trials in Which Results Would Lose Significance Under Different Assumptions on the Outcomes of LTFU Participants in Intervention and Control Arms

N=91	RI_LTFU/FU (Control)_ a			
	3	2	1.5	1
RI_LTFU/FU (intervention)_ a				
1	3	3	2	4
1.5	3	2	3	4
2	4	3	4	12
3	3	9	10	16

Among the 91 trials, percentages of results that would lose significance under less plausible assumptions: (1) none of the LTFU participants had the event, 4%; (2) all the LTFU participants had the event, 11%; (3) none of those lost to follow‐up in the treatment group had the event, and all those lost to follow‐up in the control group did (best case scenario), 3%; (4) all participants lost to follow‐up in the treatment group had the event, and none of those in the control group did (worst case scenario), 33%. FU indicates follow‐up; LTFU, loss to follow‐up.

a RI_LTFU/FU_ is the relative event incidence among those with LTFU compared with those followed up.

Results of the prespecified sensitivity analysis on the subspecialties are reported (interventional cardiology versus others) in the online Data Supplement. There was a significant difference in the proportion of trials losing significance between interventional cardiology and other subspecialties (difference, 4.35% [95% CI, 0.295%–8.41%]; *P*=0.0369; Figure S1 and Table S2).

## Discussion

We found considerable variation in the extent and reporting of LTFU data in contemporary cardiovascular clinical trials. We observed that certain characteristics of clinical trials—notably, inadequate or unclear allocation concealment, length of follow‐up, sample size, and type of intervention—were associated with increased odds of LTFU. Importantly, the primary result in 1 of 6 trials might change if reasonable assumptions were made about the end point in patients with LTFU.

The inverse‐proportion relationship noted in Figure 6 suggests that the impact of a small proportion of LTFU might be overlooked by investigators. More than one third of trials did not achieve effective blinding among either the participants or the site investigators. This finding is important because ineffective blinding is associated with overestimation of true treatment effects.^135^ Allocation concealment was inadequate or unclear in more than half of the trials. Conversely, an intention‐to‐treat analysis was used in 75% of trials, which minimizes the effects of attrition bias.^3^ Just over half of the trials included an explicit statement about LTFU, and >70% of the trials included a CONSORT flow diagram. Notably, baseline demographics on the LTFU participants were limited. Authors (93%) commonly omitted discussion of the potential impact or reasons for LTFU. We suggest that information on participants with LTFU should be included by authors in an appendix or in a defined column in a table of the trial participants’ characteristics (Table 5). Inverse probability weighting can be a helpful way of handling LTFU participants’ data, but it is not used in any of the included trials. Most trials did not impute data for LTFU participants. We noted a significant association between inadequate or unclear allocation concealment and increased odds of LTFU. This could be explained by less stringently implemented processes in trials with inadequate or unclear allocation concealment, including suboptimal measures for following up participants.

**Table 5:** Summary of the Important Common Issues for LTFU and Guidance in Conducting Trials and Reporting Trial Results

Issues That Should Be Noted	Guidance
Inadequate or unclear allocation concealment	If allocation concealment forms part of the trial design, then effective approaches to achieve allocation concealment include using a matched placebo (visually identical to the active treatment); central randomization (performed at a site remote from the trial's location); sequentially numbered, sealed, opaque envelopes^3^
Large sample size and long follow up duration	LTFU increases with larger trial sample size, hence investigators should be aware and mitigate the number of LTFU for increase in sample size and 1‐y increase in duration
Reporting of LTFU	Investigators should strive to reduce the number of LTFU. A CONSORT diagram should be included for readers. When LTFU has occurred, baseline characteristics, and extent of follow up duration before exclusion should be reported in the manuscript or supplement. The implications of LTFU should also be discussed in the manuscript. Time of dropout can be noted on a supplement or in the result paragraph or on the CONSORT diagram for readers to know the extent of follow up before dropout

CONSORT indicates Consolidated Standards of Reporting Trials; LTFU, loss to follow‐up.

### Strengths and Weaknesses of the Study

Our study has several strengths. First, the forms for screening of the trials and related data collection were established before the start of the data collection process. In addition, the calibration exercise was completed upfront as a preparatory step intended to increase accuracy for the screening and data collection. Second, a range of assumptions was made for the participants with LTFU and explored the potential effect of LTFU on the estimate of the effect of the intervention, including whether or not the trial met statistical significance on its primary outcome and the change in the relative risk ratio and number of outcome events. The effect is focused on cardiovascular trials. Our analysis depends on the accuracy and clarity of the included reports. Generalizability is also an issue. We focused our analysis on 8 journals’ publications during a 5‐year period (2014–2018). A wider inclusion strategy with more journals (with lower impact factors) and trials with a nonsignificant primary outcome result might have returned different results. Our findings might underestimate the true effect of LTFU in the effect estimate if a wider range of RCTs were included. Our review included trials with binary data only because of the design of the review analysis, which might further weaken the generalizability of the results. Time of dropout can be a factor influencing the LTFU effect because early dropouts can influence the result to a larger extent than late dropouts. However, exact time of dropout is not noted in the reports, and we are unable to stratify the effect.

### Implications

Investigators and sponsors should strive to reduce the number of participants with LTFU. The higher the LTFU, the more uncertainty increases around the treatment effect estimate and the potential for a false result. In the unfortunate event that LTFU happened, its impact can be estimated using the worst assumption (Data S7). As for the reporting of LTFU, editors may consider requiring authors to provide a fully informative and transparent report on participant LTFU including the inclusion and exclusion criteria of patients, which is in line with CONSORT guidelines. Specifically, investigators should provide information on participants with LTFU including their baseline characteristics, reasons for LTFU, and duration of follow‐up before exclusion and then compared with those who completed follow‐up. This information could be published as an appendix. Implications of LTFU should be discussed when LTFU has occurred (Table 5). This review provides estimates of the probability that the primary analysis of cardiovascular trials could lose statistical significance when LTFU events are taken into account by making appropriate estimate of event incidence. Although the 4 less plausible but commonly used assumptions may not eventuate, they can be taken as the upper limit of change in trial significance. Early LTFU has a more influential effect on the analysis than late LTFU near the overall study duration, which highlighted the need for investigators to stratify LTFU by follow‐up extent. Future studies can look at the extent of change in treatment effect in relation to the LTFU proportion and event number and the effect of partial and full LTFU defined as difference in the extent of follow‐up before exclusion. The influence of dropout time on LTFU effect can be explored for assessing the possibility of systemic inclusion of patients accounting for early dropouts.

## Conclusions

Almost 1 in 6 (16%) cardiovascular randomized trials published in leading journals may have a change in the primary outcome if plausible assumptions are made about differential event rates of participants lost to follow‐up. There is scope for improvement arising from LTFU in randomized trials in cardiovascular medicine. Bias minimization through mitigation of participants lost to follow‐up offers the opportunity to enhance the value of randomized trials.

## Sources of Funding

The study was funded by British Heart Foundation (PG/17/2532884; RE/13/5/30177; RE/18/6134217). The funder had no role in the study design, the writing of manuscript, or the decision to submit this article or future manuscripts for publication.

## Disclosures

Berry is employed by the University of Glasgow which holds consultancy and research agreements for his work with companies that have commercial interests in the diagnosis and treatment of angina. The companies include Abbott Vascular, Astra Zeneca, Boehringer Ingelheim, GSK, HeartFlow, Menarini, Novartis, and Siemens Healthcare. Jüni serves as unpaid member of the steering group of trials funded by Astra Zeneca, Biotronik, Biosensors, St. Jude Medical and The Medicines Company, has received research grants to the institution from Astra Zeneca, Biotronik, Biosensors International, Eli Lilly and The Medicines Company, and honoraria to the institution for participation in advisory boards from Amgen, but has not received personal payments by any pharmaceutical company or device manufacturer. The remaining authors have no disclosures to report.

## Supporting information


**Datas S1–S7 Tables S1–S2 Figure S1 References 14, and 17–134**
