# Supplementary material for: Bias and Loss to Follow‐Up in Cardiovascular Randomized Trials: A Systematic Review
Source: J Am Heart Assoc. 2020 Jul 9;9(14):e015361. doi: 10.1161/JAHA.119.015361 (PMC7660731; doi:10.1161/JAHA.119.015361)
Supplement: Supplementary file 1 — Datas S1–S7 Tables S1–S2 Figure S1 References 14, and 17–134 [file JAH3-9-e015361-s001.pdf]

SUPPLEMENTAL MATERIAL

Data S1 - Hierarchy of outcomes relative to patient importance in FLUKE

I. Mortality

   a. All-cause mortality

   b. Disease specific mortality

II. Morbidity

   a. Cardiovascular major morbid events

   b. Other major morbid events (e.g. Revascularization)

   c. Onset/recurrence/relapse/remission of diseases

   d. Hospitalization, medical and surgical procedures

   e. Infections

Data S2- Search strategy for Medline and Embase using OVID interface

Medline

1 exp Myocardial Ischemia/

2 (MYOCARD$4 adj4 (ISCHAEMI$2 or ISCHEMI$2)).tw.

3 exp Coronary Artery Bypass/

4 ((ISCHAEMI$2 or ISCHEMI$2) adj4 HEART).tw.

5 CORONARY.ti,ab.

6 exp Coronary Disease/

7 exp Myocardial Revascularization/

8 exp Myocardial Infarction/

9 (MYOCARD$5 adj4 INFARCT$5).tw.

10 (HEART adj4 INFARCT$5).tw.

11 exp Angina Pectoris/

12 ANGINA.tw.

13 exp Heart Failure/

14 (HEART adj6 Failure).tw.

15 or/1-14

16 exp Heart Diseases/

17 (Heart adj4 disease$2).tw.

18 MYOCARD$5.tw.

19 CARDIAC$2.tw.

20 CABG.tw.

21 PTCA.tw.

22 (STENT$4 and HEART).tw.

23 Heart Bypass, Left/ or Heart Bypass, Right/

24 CARDIOLOGY SERVICE, HOSPITAL/ or CARDIOLOGY/

25 or/16-24

26 15 or 25

27 Randomized controlled trial.pt.

28 randomized controlled trial/

29 (random$ or placebo$).ti,ab,sh.

30 ((singl$ or double$ or triple$ or treble$) and (blind$ or mask$)).tw,sh.

31 or/27-30

32 (retraction of publication or retracted publication).pt.

33 31 or 32

34 (ANIMALS not HUMANS).sh.

35 33 not 34

36 35 and 26

37 bmj.jn

38 “Annals of Internal Medicine”.jn.

39 jama.jn.

40 lancet.jn.

41 “new england journal of medicine”.jn.

42 36 and 37

43 36 and 38

44 36 and 39

45 36 and 40

46 36 and 41

47 european heart journal.jn.

48 circulation.jn.

49 journal of the American college of cardiology.jn.

50 36 and 47

51 36 and 48

52 36 and 49

53 42 or 43 or 44 or 45 or 46 or 50 or 51 or 52

54 limit 53 to yr=”2014-2018”

EMBASE

1 Heart Disease/

2 (MYOCARD$4 adj2 (ISCHAEMI$2 or ISCHEMI$2)).tw.

3 ((ISCHAEMI$2 or ISCHEMI$2) adj4 HEART).tw.

4 Coronary Artery Disease/

5 Transluminal Coronary Angioplasty/

6 (CORONARY adj4 (DISEASE$2 or BYPASS$2 or THROMBO$5 or ANGIOPLAST$2)).tw.

7 Heart Infarction/

8 (MYOCARD$4 adj2 INFARCT$5).tw.

9 (HEART adj2 INFARC$5).tw.

10 Heart Muscle Revascularization/

11 Angina Pectoris/

12 ANGINA.tw.

13 (HEART adj2 FAILURE).tw.

14 (HEART adj2 DISEASE$2).tw.

15 CARDIAC$2.tw.

16 CABG.tw.

17 PTCA.tw.

18 (STENT$4 and HEART).ti,ab.

19 Extracorporeal Circulation/

20 cardiology/

21 or/1-20

22 Randomized Controlled Trial/

23 Single Blind Procedure/

24 Double Blind Procedure/

25 Crossover Procedure/

26 22 or 23 or 24 or 25

27 (random$ or factorial$ or crossover$ or placebo$ or (cross adj over) or assign$).ti,ab.

28 ((singl$ or double$ or triple$ or treble$) and (blind$ or mask$)).ti,ab.

29 controlled clinical trial*.ti,ab.

30 28 or 26 or 27 or 29

31 21 and 30

32 (animal$ not human$).sh,hw.

33 31 not 32

34 bmj.jn

35 “Annals of Internal Medicine”.jn.

36 jama.jn.

37 lancet.jn.

38 “new england journal of medicine”.jn.

39 european heart journal.jn.

40 circulation.jn.

41 journal of the American college of cardiology.jn.

42 33 and 34

43 33 and 35

44 33 and 36

45 33 and 37

46 33 and 38

47 33 and 39

48 33 and 40

49 33 and 41

50 or/42-46

51 or/47-49

52 limit 51 to article or article in press or conference paper

53 50 or 52

54 limit 53 to yr =”2014-2018”

Data S3 – FLUKE study data screening form

FLUKE study: ***Data Screening Form***

Screener initials: **Study ID:** **Author, year: ________________,**
Journal: ☐ AIM ☐ BMJ ☐ JAMA ☐ Lancet ☐ NEJM

1. **Eligible RCT Report?** ☐ No ☐ **Exclude** → *stop here*
   ☐ Yes, *type of RCT:*
   ☐ Two arms ☐ Multiple Arms
   ☐ Factorial design

2. **Trial described as:** ☐ Non-inferiority
   ☐ Equivalence
   ☐ Neither

3. **Primary outcome clearly specified.** ☐ Yes, one: ______________________________ *(go to q5)*
   ☐ No, multiple primary outcomes: ________________ *(go to q4)*
   ☐ None specified *(go to q4)*

4. **If multiple or no primary outcome specified, select one : ______**

5. **Primary outcome category #** *(refer to the guide)*: __________________________ *(e.g. II.3)*

6. **Effect on primary endpoint reported as:** ☐ Continuous outcome exclusively ☐ **Exclude**
   ☐ Multinomial outcome exclusively ☐ **Exclude**
   ☐ Binary outcome expressed as rate exclusively ☐ **Exclude**
   ☐ Binary outcome, data not available for 2x2 table ☐ **Exclude**
   ☐ Binary outcome, data available for 2x2 table, *go to the next question*

7. **Is it a composite endpoint?** ☐ Yes, *list components*:______________________________
   ☐ No

8. **Is it a patient important outcome?**
   ☐ No ☐ **Exclude**
   ☐ Yes, *go to the next question*

9. **Result statistically significant?** ☐ No ☐ **Exclude**
   ☐ Yes

Please fill out this box for each study

☐ **Include in FLUKE**

☐ **Exclude from FLUKE**

☐ **3rd reviewer needed**
(no consensus between 2 reviewers)

If exclude, reason for exclusion:
☐ Not RCT
☐ Not eligible RCT
☐ Data for the primary endpoint not available for 2x2 table
☐ Outcome not patient important
☐ Result not statistically significa

Data S4 – FLUKE study data abstraction form

FLUKE study: ***Data Abstruction Form***

Screener initials: **Study ID:** **Author, year: ________________,**
Journal: ☐ AIM ☐ BMJ ☐ JAMA ☐ Lancet ☐ NEJM

1. **Eligible RCT Report?** ☐ No ☐ **Exclude** → *stop here*
   ☐ Yes, *type of RCT:*
   ☐ Two arms ☐ Multiple Arms
   ☐ Factorial design

2. **Trial described as:** ☐ Non-inferiority
   ☐ Equivalence
   ☐ Neither

3. **Primary outcome clearly specified.** ☐ Yes, one: ________________________________ *(go to q5)*
   ☐ No, multiple primary outcomes: ________________ *(go to q4)*
   ☐ None specified *(go to q4)*

4. **If multiple or no primary outcome specified, select one : ______**

5. **Primary outcome category #** *(refer to the guide)*: ______________________________ *(e.g. II.3)*

6. **Effect on primary endpoint reported as:** ☐ Continuous outcome exclusively ☐ **Exclude**
   ☐ Multinomial outcome exclusively ☐ **Exclude**
   ☐ Binary outcome expressed as rate exclusively ☐ **Exclude**
   ☐ Binary outcome, data not available for 2x2 table ☐ **Exclude**
   ☐ Binary outcome, data available for 2x2 table, *go to the next question*

7. **Is it a composite endpoint?** ☐ Yes, *list components*:________________________________
   ☐ No

8. **Is it a patient important outcome?**
   ☐ No ☐ **Exclude**
   ☐ Yes, *go to the next question*

9. **Result statistically significant?** ☐ No ☐ **Exclude**
   ☐ Yes

Please fill out this box for each study

☐ **Include in FLUKE**

☐ **Exclude from FLUKE**

☐ **3rd reviewer needed**
(no consensus between 2 reviewers)

If exclude, reason for exclusion:
☐ Not RCT
☐ Not eligible RCT
☐ Data for the primary endpoint not available for 2x2 table
☐ Outcome not patient important
☐ Result not statistically significant

Background Information		
**10.**	**Mean/Median Age**<br>**Number of study centers**	**Age=**<br>**N=**
**11.**	**Funding**<br>*Check all that apply*	☐ Private only for profit, other<br>☐ Private not fro profit<br>☐ Government<br>☐ Not funded<br>☐ Not reported
**12.**	**Clinical Area**<br>*Check only one*	☐ Medical<br>☐Pharmacological<br>☐ Surgical<br>☐Electrophysiology<br>☐Heart failure<br>☐Interventional cardiology<br>☐Open heart surgery<br>☐General cardiology<br>☐Cardiovascular imaging<br>☐ Others
**13.**	**Intervention**<br>*Check only one*	☐ Pharmacological<br>☐ Surgery<br>☐ Rehabilitation<br>☐ Behavioral intervention<br>☐ Complementary and alternative medicine<br>☐ Diagnostic test<br>☐ Other (specify)
**14.**	**Control**<br>*Check only one*	☐Standard care<br>☐Placebo<br>☐Pharmacological<br>☐Surgery<br>☐Rehabilitation<br>☐Behavioral intervention<br>☐Diagnostic test<br>☐Other (specify)

Methodological Quality		
**15.**	**Concealment of Allocation**<br>Check only one	☐ Adequate (involving the use of sequentially numbered, opaque, sealed envelope or coded medication containers or central randomization or quasi-randomized)<br>☐ Inadequate (Like Open random allocation schedule)<br>☐No method described<br>☐Not concealed<br>☐Not reported
**16.**	**Blinding of patients**	☐Adequate<br>☐Inadequate<br>☐Not reported
**17.**	**Blinding of health care providers**	☐Adequate<br>☐Inadequate<br>☐Not reported
**18.**	**Blinding of data collectors**	☐Adequate<br>☐Inadequate<br>☐Not reported
**19.**	**Blinding of outcome adjudicators**	☐Adequate<br>☐Inadequate<br>☐Not reported
**20.**	**Blinding of data analysts**	☐Adequate<br>☐Inadequate<br>☐Not reported
**21.**	**Study stopped early for benefit**	☐Yes<br>☐No

ITT Principle		
**22.**	**Authors used the term ITT**	☐ Yes, ITT<br>☐ Yes, Modified ITT<br>☐ No
**23.**	**Post randomization exclusion of mistakenly randomized**	☐Yes (Skip Question 24 and 25)<br>☐No (Go to question 24 and 25)<br>☐Not reported
**24.**	Information about ineligibility was available at randomization	☐Yes<br>☐No<br>☐Not reported
**25.**	Post randomization exclusions were blinded to allocation	☐Yes<br>☐No<br>☐Not reported
**26.**	**Patients for whom outcome data is available were analyzed in the arm to which they were randomized**	☐Yes<br>☐No<br>☐Not reported

LTFU statements		
27.	**LTFU explicitly reported**	☐ Explicit statement: LTFU occurred<br>☐ Explicit statement: LTFU did not occur<br>☐ No explicit statement about LTFU
28.	**CONSORT flow diagram**	☐CONSORT diagram showing LTFU<br>☐CONSORT diagram not showing LTFU<br>☐No CONSORT diagram
29.	**For studies with no explicit statement about LTFU and no consort diagram**	☐Meet all 3 prespecified criteria<br>☐ Does not meet all 3 prespecified criteria<br>☐N/A
30.	**LTFU reported separately for the 2 arms**	☐Yes<br>☐No
31.	**Authors compared baseline characteristics of LTFU**	☐Yes<br>☐No
32.	**Implications of LTFU discussed**	☐Yes<br>☐No
33.	**Methods of dealing with LTFU explicitly described**	☐Yes, methods<br>☐Yes, results<br>☐No

Methods of dealing with LTFU		
34	**Methods**	
	Not applicable, no LTFU occurred	☐
	Not applicable, uncertain whether LTFU occurred	☐
	Unclear which method used	☐
	Survival analysis	☐
	Complete case analysis	☐
	Worst case scenario	☐
	Best case scenario	☐
	None of the LTFU had the outcome	☐
	All the LTFU had the outcome	☐
	Different methods for different subgroups of LTFU	☐
	Other (specify)	☐

LTFU statistical data					
Primary outcome data		Intervention	control	total	Prespecified assumptions for different groups of LTFU
35.	**Mistakenly randomized, inappropriately excluded (subtotal 1)**				
36.	**Did not receive intervention, inappropriately excluded (subtotal 2)**				
37.	Withdrew consent *(subtotal 3)*<br>☐unclear whether followed up<br>☐not followed up<br>☐followed up, not included in the analysis *(not LTFU for FLUKE)*				
38.	**Withdrew consent due to side effect or adverse event**				
39.	**Withdrew consent due to other specified reason**				
40.	**Withdrew consent due to unclear reason**				
41.	Cross over *(subtotal 4)*<br>☐unclear whether followed up<br>☐not followed up<br>☐followed up, not included in the analysis *(not LTFU for FLUKE)*<br>☐followed up, analyzed in a group not randomized to *(not LTFU for FLUKE)*				
42.	**Cross over due to side effect or adverse event**				
43.	**Cross over due to other specified reason**				
44.	Cross over due to unclear reason				
45.	Non adherent *(subtotal 5)*<br>☐unclear whether followed up<br>☐not followed up<br>☐followed up, not included in the analysis *(not LTFU for FLUKE)*<br>☐followed up, analyzed in a group not randomized to *(not LTFU for FLUKE)*				
46.	**Non adherent due to side effect or adverse event**				
47.	**Non adherent due to other specified reason**				
48.	Non adherent due to unclear reason				
49.	Lost contact and no other source of outcome data				

**50.**	Others				
**51.**	LTFU total				
**LTFU statistical data**					
**Primary outcome data**		**Intervention**	**control**	**total**	
**52.**	**Mistakenly randomized, inappropriately excluded (subtotal 1)**				
**53.**	**Did not receive intervention, inappropriately excluded (subtotal 2)**				
**54.**	**Randomized**				
**55.**	**Randomized adjusted (54-53-52)**				
**Primary outcome data**		**Intervention events**	**Control events**		
**39.**	**Included in primary analysis**				
**40.**	**Unadjusted effect estimate;95% CI; P value**				

Data S5: Further elaboration on the methodology adopted in the systematic review

Analysis method

a. Assessment on the methodological and reporting quality

Bikdeli et al first reported a set of risk factors to consider when evaluating methodological and reporting quality of trials in 2019.[14] We consider limiting factors to include the following:

1. Inadequate allocation sequence concealment
2. No blinding on patient
3. Early stoppage
4. Not using intention-to-treat analysis
5. Absence of protocol
6. Without explicit statement on the status of loss to follow up

A univariable random-effects meta-regression analysis was conducted using the log odds of participants lost to follow-up as the dependent variable and general trial characteristics and methodological characteristics as independent variables

1. General trial characteristics
    a. Number of centres
    b. Sample size
    c. Length of follow-up
    d. Type of intervention (Surgery/interventional vs other)
    e. Cardiology Subspecialty (General Medical vs Others)
    f. Type of funding (Commercial Vs Non-profit organisations, governmental or none)
2. Methodological trial characteristics
    a. Concealment of allocation
    b. Blinding of patients
    c. Stopping early for benefit
    d. Use of intention to treat analysis

b. Extent of loss to follow-up

The extent of LTFU was estimated by calculating the percentage of LTFU in each trial from each arm (intervention and control). Then, median and interquartile range of the percentages across trials were obtained. A ratio of the total number of participants identified as LTFU to the number of primary outcome events was calculated for each trial (the "lost to follow-up to events ratio"). Median and standard deviation of this ratio was also calculated across the trials.

c. Potential impact of loss to follow-up

The potential impact of LTFU is evaluated by proposing assumptions about the outcomes of participants LTFU and the estimated effect of that assumption on the primary outcome (Data S6 for examples). The following common assumptions are first used for calculation:

a. None of the participants lost to follow-up had the event
b. All the participants lost to follow-up had the event
c. None of those lost to follow-up in the treatment group had the event and all those lost to follow-up in the control group did (best case scenario)
d. All participants lost to follow-up in the treatment group had the event and none of those in the control group did (worst case scenario)

Although the above assumptions are widely used in multiple literatures, some experts have countered they are not plausible and have suggested a novel method for estimating effects of LTFU.[17] Akl et al evaluated more plausible assumptions that the incidence of events among participants lost to follow-up is higher by a specific ratio relative to the observed event incidence among participants followed up. [17] They defined $RI_{LTFU/FU}$ as the event incidence among those lost to follow-up relative to the event incidence among those followed up and made plausible assumptions towards the outcome of LTFU participants (see data S6). LTFU refers to "lost to follow-up" and FU refers to "followed up". A range of plausible $RI_{LTFU/FU}$ values (1,1.5,2,3) was used in both the intervention group and the control group. 3 is the upper limit, which was previously determined by consensus. [17]

Data S6 - Illustrations of the assumptions being considered in FLUKE with examples

Examples based on the following theoretical trial:

- Randomization: 100 to intervention while 100 to control group
- Loss to follow up: 20 in the intervention group while 10 in the control group
- Events: 15 in the intervention group while 20 in the control group

Assumption 1: None of the lost to follow-up participants had the event

		intervention	Control
a	Lost to follow up	20	10
b	Events assumed*	0	0
c	Events observed in the trial	15	20
d	Total events (b+c)	15	20
e	Randomized	100	100
f	Risk (d/e)	0.15	0.2
g	Relative risk	0.75	

**None of the lost to follow-up in either group had an event*

Assumption 2: All lost to follow-up participants had the event

		intervention	Control
a	Lost to follow up	20	10
b	Events assumed*	20	10
c	Events observed in the trial	15	20
d	Total events (b+c)	35	30
e	Randomized	100	100
f	Risk (d/e)	0.35	0.3
g	Relative risk	1.17	

**Each of the lost to follow-up both groups had an event*

Assumption 3: Best case scenario

		intervention	Control
a	Lost to follow up	20	10
b	Events assumed*	0	10
c	Events observed in the trial	15	20
d	Total events (b+c)	15	30
e	Randomized	100	100
f	Risk (d/e)	0.15	0.3
g	Relative risk	0.5	

** None of those lost to follow up in the treatment group had the event and all those lost to follow up in the control group did*

Assumption 4: Worst case scenario

		intervention	Control
a	Lost to follow up	20	10
b	Events assumed*	20	0
c	Events observed in the trial	15	20
d	Total events (b+c)	35	20
e	Randomized	100	100
f	Risk (d/e)	0.35	0.2
g	Relative risk	1.75	

** All participants lost to follow up in the treatment group had the event and none of those in the control group did*

Assumptions using relative event incidence ($RI_{LTFU/FU}$)

$RI_{LTFU/FU}$ refers to the event incidence among those lost to follow-up (LTFU) relative to the event incidence among those followed up (FU)

$RI_{LTFU/FU}$ = (Number of events among LTFU / number of LTFU) / (Number of events among FU / number of FU)

- $RI_{LTFU/FU}$ =1; the event incidence among LTFU and FU is equal
- $RI_{LTFU/FU}$ >1; the event incidence among LTFU is greater than that of FU

Assumption 1: $RI_{LTFU/FU}$ = 1 in intervention group; and $RI_{LTFU/FU}$ = 3 in control group

		intervention	Control
a	Lost to follow up	20	10
b	Events assumed*	(20)(1)(15/80)=4	(10)(3)(20/90)=7
c	Events observed in the trial	15	20
d	Total events (b+c)	19	27
e	Randomized	100	100
f	Risk (d/e)	0.19	0.27
g	Relative risk	0.70	

** Number of events assumed= (number lost to follow up) x ($RI_{LTFU/FU}$) x (Events observed/(number randomized – number lost to follow up))*

Assumption 2: $RI_{LTFU/FU}$ = 3 in intervention group; and $RI_{LTFU/FU}$ = 1.5 in control group

		intervention	Control
a	Lost to follow up	20	10
b	Events assumed*	(20)(3)(15/80)=11	(10)(1.5)(20/90)=3
c	Events observed in the trial	15	20
d	Total events (b+c)	26	23
e	Randomized	100	100
f	Risk (d/e)	0.26	0.23
g	Relative risk	1.13	

** Number of events assumed= (number lost to follow up) x ($RI_{LTFU/FU}$) x (Events observed/(number randomized – number lost to follow up))*

Data S7 – Estimation method accounting for LTFU

Assumptions made using relative event incidence ($RI_{LTFU/FU}$)

$RI_{LTFU/FU}$ refers to the event incidence among those lost to follow-up (LTFU) relative to the event incidence among those followed up (FU) *

Worst $RI_{LTFU/FU}$ assumption = 3 in intervention arm 1 in control arm

Assumption: $RI_{LTFU/FU}$ = 3 in intervention group; and $RI_{LTFU/FU}$ = 1 in control group *

		Intervention	Control
a	Number of Lost to follow up	X	Y
b	Event Rate (ER)	(Intervention Event / Number of participants in intervention)	(Control Event/ Number of participants in control)
c	Events assumed†	(X)(3)(ER_{inter}) = m	(Y)(1)(ER_{contr}) = n
d	Events observed in the trial	Intervention Event	Control Event
e	Total events (c+d)	m + Intervention Event	n + Control Event
f	Randomized	Number of participants in intervention	Number of participants in control
G	Risk (e/f)	R_{inter}	R_{contr}
h	Relative risk	R_{inter} / R_{contr}	

** $RI_{LTFU/FU}$ = (Number of events among LTFU / number of LTFU) / (Number of events among FU / number of FU)*

- *$RI_{LTFU/FU}$ =1; the event incidence among LTFU and FU is equal*
- *$RI_{LTFU/FU}$ >1; the event incidence among LTFU is greater than that of FU*

† Number of events assumed= (number lost to follow up) x ($RI_{LTFU/FU}$) x (Events observed/(number randomized – number lost to follow up))

Data S8 – Among the 23 trials from intervention cardiology, percentage which results would lose significance under different assumptions:

- No events experienced by any lost to follow-up participants =0%
- Events experienced by all the lost to follow-up participants =17%
- Events only experienced by the LTFU in control group while no events experienced by the LTFU in treatment group (best case scenario) =0%
- Events only experienced by the LTFU in treatment group while no events experienced by the LTFU in control group (worst case scenario) =22%

Among the 68 trials from other cardiology field, percentage which results would lose significance under different assumptions:

- No events experienced by any lost to follow-up participants =6%
- Events experienced by all the lost to follow-up participants =9%
- Events only experienced by the LTFU in control group while no events experienced by the LTFU in treatment group (best case scenario) =4%
- Events only experienced by the LTFU in treatment group while no events experienced by the LTFU in control group (worst case scenario) =37%

Table S1 – Reference list of the 117 studies included in FLUKE

Study reference	Country	Journal	Mean age
S. Verheye (2015) [123]	Belgium	NEJM	67.8
S. S. Anand (2018) [20]	Canada	The Lancet	67.8
M. Dewey (2016) [42]	Germany	BMJ	60.4
HPS/TIMI55- REVEAL Group (2017) [28]	UK	NEJM	67
ASCEND Study Group (2018) [29]	UK	NEJM	63.2
H. Calkins (2017) [32]	Germany	NEJM	59.2
C. P. Cannon (2017) [33]	USA	NEJM	70.8
Stuart J Connolly (2018) [37]	Canada	The Lancet	68.3
P J Devereaux (2018) [41]	Canada	The Lancet	70
J.W. Eikelboom (2018) [48]	Canada	NEJM	68.2
R. Estruch (2018) [50]	Spain	NEJM	67
C. M. Gibson (2016) [54]	USA	NEJM	70.1
E. J. Velazquez (2016) [122]	USA	NEJM	59.5
J. P. Greenwood (2016) [57]	UK	JAMA	56.3
Q. Zhao (2018) [133]	China	JAMA	63.6
B. P Halliday (2018) [59]	UK	The Lancet	55
A. F. Hernandez (2018) [62]	USA	The Lancet	64.1
S. C. Johnston (2018) [69]	USA	NEJM	65
W.N. Kernan (2016) [73]	USA	NEJM	63.5
JM. Kim (2018) [74]	South Korea	JAMA	60
S. Yusuf (2016) [129]	Canada	NEJM	65.7
N. F. Marrouche (2018) [78]	USA	NEJM	64
S. P. Marso (2016) [79]	USA	NEJM	64.3
J. L. Mas (2017) [80]	France	NEJM	43.7
M. S. Maurer (2018) [82]	USA	NEJM	75
D. E Kandzari (2017) [72]	USA	The Lancet	64.5

M.R. Mehra (2018) [84]	USA	NEJM	60
M. R. Mehra (2016) [85]	USA	NEJM	59.6
M.E. Wechsler (2017) [124]	USA	NEJM	48.5
A. N Patel (2016) [92]	USA	The Lancet	65
G.D. Perkins (2018) [93]	UK	NEJM	69.7
S. R. Steinhubl (2018) [110]	USA	JAMA	72.3
P.M. Ridker (2017) [97]	USA	NEJM	61
M. Valgimigli (2015) [119]	Netherlands	The Lancet	65.8
M. S. Sabatine (2017) [100]	USA	NEJM	63
J. L. Sapp (2016) [102]	Canada	NEJM	68.6
J. L. Saver (2017) [104]	USA	NEJM	45.9
G.G. Schwartz, (2018) [105]	USA	NEJM	58.6
P. C. Smits (2017) [107]	Netherlands	NEJM	61.3
B. Zinman (2015) [134]	Canada	NEJM	63.1
L. Søndergaard (2017) [109]	Denmark	NEJM	45.2
G.W. Stone (2018) [111]	USA	NEJM	72.3
N. Tegn (2016) [114]	Norway	The Lancet	84.8
H. Thiele (2017) [115]	Germany	NEJM	70
M. Valgimigli (2018) [118]	Switzerland	The Lancet	65.8
O. Varenne (2017) [121]	France	The Lancet	81.4
S. Yusuf (2016) [128]	Canada	NEJM	65.7
A. Zarbock (2015) [130]	Germany	JAMA	70.4
S.D. Wiviott (2018) [125]	USA	NEJM	79.9
M. Abdel-Wahab (2014) [18]	Germany	JAMA	80.8
D. H. Adams (2014) [19]	USA	NEJM	83.3
A. Appelboam (2015) [21]	UK	The Lancet	54.8
M. P. Bonaca (2015) [25]	USA	NEJM	65.3
S. S Brar (2014) [30]	USA	The Lancet	71.5
C. P. Cannon (2015) [34]	USA	NEJM	63.6

B. De Bruyne (2014) [40]	Belgium	NEJM	63.7
J. D. Douketis (2015) [47]	USA	NEJM	71.7
T. Engstrøm (2015) [49]	Denmark	The Lancet	63.5
A. M. Gillinov (2015) [55]	USA	NEJM	69.6
D. J. Gladstone (2014) [63]	Canada	NEJM	72.8
THE SPRINT Research Group (2015) [126]	USA	NEJM	67.9
Y. Han (2015) [60]	China	JAMA	57.7
G. Hindricks (2014) [63]	Germany	The Lancet	65.5
SJ Hong (2015) [64]	Korea	JAMA	64
Y. Huo (2015) [65]	China	JAMA	60
M. Imazio (2014) [66]	Italy	The Lancet	48.8
M. Imazio (2014) [67]	Italy	JAMA	67.5
J. J. McMurray (2014) [83]	UK	NEJM	63.8
G. Meyer (2014) [86]	Germany	NEJM	66.1
C. A. Morillo (2014) [89]	Canada	JAMA	55.3
V. Y. Reddy (2014) [96]	USA	JAMA	72
M. Ringh (2015) [99]	Sweden	NEJM	72.4
T. Sanna (2014) [101]	Italy	NEJM	61.5
A. Shahzad (2014) [106]	UK	The Lancet	63.3
P. Urban (2015) [117]	Switzerland	NEJM	75.7
F. Tomai (2014) [116]	Italy	circulation	73
M. Valgimigli (2015) [120]	Netherlands	JACC	71.8
X. D. Zhang (2014) [132]	China	European Heart Journal	59.2
I. Taguchi (2018) [112]	Japan	circulation	68.1
Y. D. Tang (2018) [113]	china	circulation	58.5
J. Zhang (2018) [131]	china	JACC	65.6
J. Bermejo (2018) [22]	Spain	European Heart Journal	71.44
R. S. Bhatia (2017) [24]	Canada	JACC	68
D. Bonnet (2017) [26]	France	JACC	5.8

M. Brignole (2018) [31]	Italy	European Heart Journal	71.5
SL Chen (2017) [89]	China	JACC	64.5
T. Cuisset (2016) [38]	France	European Heart Journal	60
L. Di Biase (2016) [44]	USA	JACC	63.9
L. Di Biase (2016) [46]	USA	circulation	61
M. E. Farkouh (2018) [51]	Canada	JACC	63.1
P. Garot (2016) [52]	France	JACC	75.7
J. P.J. Halcox (2017) [58]	UK	circulation	72.6
K. Kaitani (2016) [71]	Japan	European Heart Journal	63.3
PH Lee (2018) [76]	South Korea	JACC	51.5
G. Sardella (2016) [103]	Italy	JACC	72.5
H. Sohara (2016) [108]	Japan	JACC	59.5
F. M. Notarangelo (2018) [90]	Italy	JACC	70.9
M. Ortiz (2017) [91]	Spain	European Heart Journal	65.3
J. Yang (2014) [102]	china	JACC	5.65
J. Pu (2017) [94]	China	circulation	58
M. Rienstra (2017) [98]	Netherlands	European Heart Journal	64.5
I. Bernat (2014) [23]	Czech Republic	JACC	62.1
G. Boriani (2014) [27]	italy	European Heart Journal	73.5
D. Carrick (2014) [35]	UK	JACC	59.6
A. de Belder (2014) [39]	UK	JACC	83.5
L. Di Biase (2015) [43]	USA	JACC	66
L. Di Biase (2014) [45]	USA	circulation	61.5
A. H. Gershlick (2015) [53]	UK	JACC	64.9
Y. Han (2014) [112]	China	JACC	61.4
C. Jennings (2014) [68]	UK	European Heart Journal	60
J. Layland (2015) [75]	UK	European Heart Journal	62
M. Leoncini (2014) [77]	Italy	JACC	66.2
C. Kaiser (2014) [70]	Switzerland	circulation	62.5

Y. Matsumoto (2014) [81]	Japan	JACC	67.5
L. Mont (2014) [87]	Spain	European Heart Journal	55
G. Montalescot (2014) [88]	France	European Heart Journal	58.2
M. J. Reardon (2015) [95]	USA	JACC	83.2

Table S2- Sensitivity analysis of the percentage of eligible trials on intervention cardiology vs trials on other subjects in which results would lose significance under different assumptions on the LTFU outcomes on intervention and control group

**Intervention Cardiology †**					**Others †**				
**N=23**	**RI $_{LTFU/FU\ (Control)}$ ***				**N=68**	**RI $_{LTFU/FU\ (Control)}$ ***			
	**3**	**2**	**1.5**	**1**		**3**	**2**	**1.5**	**1**
**RI $_{LTFU/FU\ (intervention)}$ ***					**RI $_{LTFU/FU\ (intervention)}$ ***				
**1**	9	9	0	0	**1**	1	1	3	6
**1.5**	9	0	0	0	**1.5**	1	3	4	6
**2**	9	0	0	0	**2**	3	5	6	16
**3**	0	0	0	0	**3**	4	12	13	22

N= Number

* RI $_{LTFU/FU}$ is the relative event incidence among those lost to follow-up compared with those followed up

† Paired T test shows that there is significant difference between the subgroup across different assumptions (Mean difference =4.35% ,95%CI 0.295%-8.41%, p=0.0369)

Figure S1- Scatterplot of the proportion of trials losing significance based on various assumption stratified by subspecialty

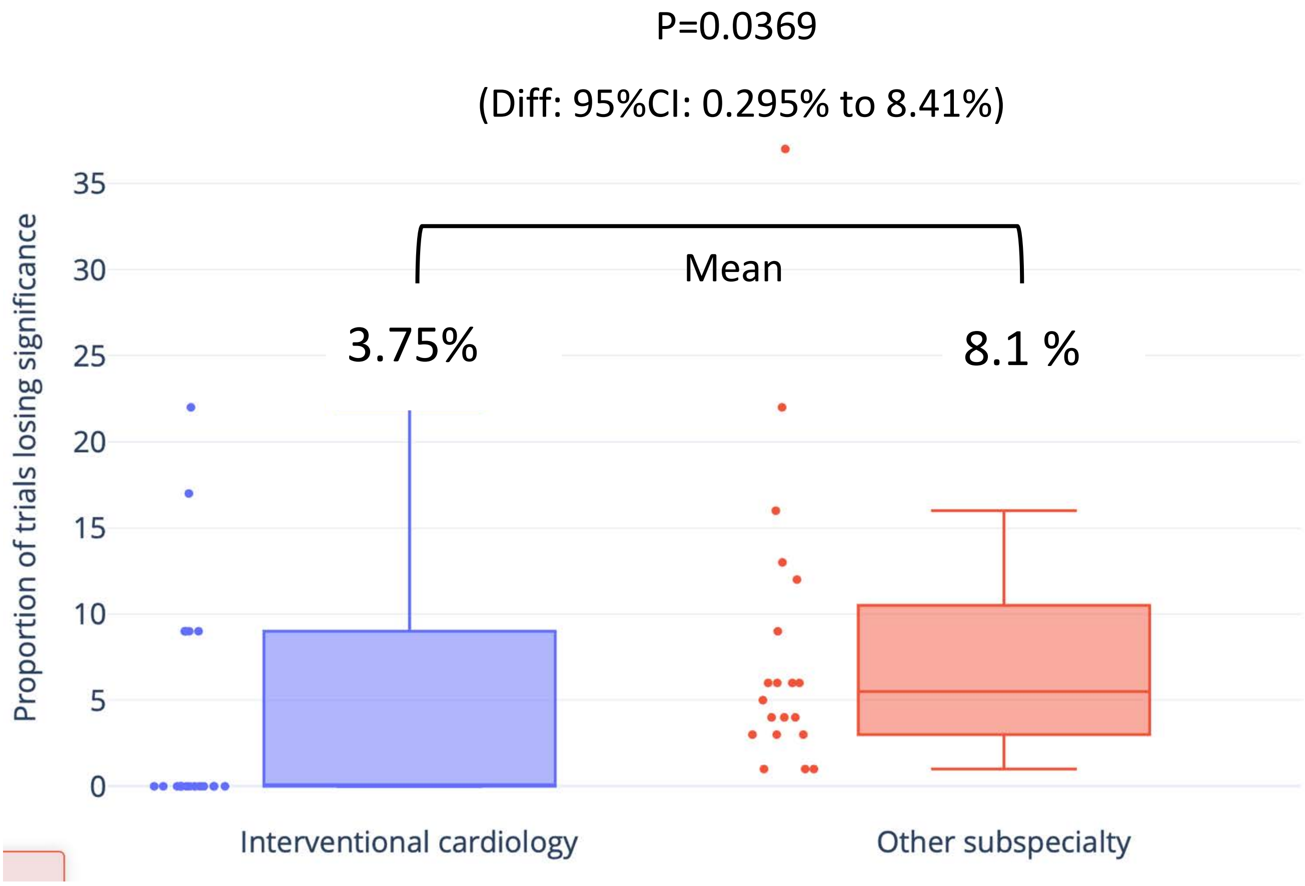

Legend- CI = Confidence Interval; Diff = Difference; LTFU = Lost to follow up; p = p-value

Figure S1 shows the proportion of trials losing significance based on each assumption. It is grouped by the different type of subspecialty. A mean of 3.75% trials form the interventional cardiology subspecialty lost significance while 8.1% trials from other subspecialty lost significance. A paired sample t test was run against the subspecialty yielding a significant difference in proportions (p-value = 0.0.0369)
